# Succession of Lichens in Front of Retreating Glaciers in Sweden and Norway

**DOI:** 10.1002/ece3.71848

**Published:** 2025-07-23

**Authors:** Josef P. Halda, Jan Košnar, Alena Lukešová

**Affiliations:** ^1^ Faculty of Science, Department of Biology University of Hradec Králové Hradec Králové Czech Republic; ^2^ Nature Conservation Agency of the Czech Republic Regional Office East Bohemia Pardubice Czech Republic; ^3^ Department of Soil Microbiology and Soil Chemistry Biology Centre CAS České Budějovice Czech Republic

**Keywords:** Arctic, biocrust, climate warming, glaciers, lichen, Scandinavia, species distribution model, succession, tundra

## Abstract

Climate change is accelerating the melting of glaciers to create new habitats colonized by pioneer organisms. Lichens are adapted to extreme conditions and, together with cyanobacteria and algae, are generally among the first organisms to appear in primary succession. The exact mechanism of lichen community formation and the factors influencing species turnover are still poorly understood. The aim was to determine the time zones of succession of terricolous and saxicolous species after glacier retreat, and to identify common ecological traits among pioneer species, including thallus type. Additionally, lichen species were categorized into time periods based on their colonization rates. Finally, differences among dated plots in the glacier forelands of Midtdalsbreen (Norway) and Storglaciären (Sweden) were compared. Taxon: Lichens. Location: Arctic. A total of 27 plots (135 subplots) were delineated across five successional age classes (9–264 years postglaciation). Lichen abundances and environmental variables (e.g., orientation, substrate composition) were recorded. Canonical correspondence analysis (CCA) and detrended correspondence analysis (DCA) were used to analyze species composition gradients and the effect of successional age. Monte Carlo permutation tests were performed to determine statistical significance (499 permutations). Lichen species richness increased rapidly in the early succession (15–25 years), but stagnated or slightly declined in older stages (> 100 years), probably due to competitive displacement. Early successional pioneer species included *Stereocaulon alpinum*, *Cetraria islandica* , and *Flavocetraria nivalis*—fruticose lichens that disperse easily by wind through thallus fragments. Late successional species, such as *Arctoparmelia centrifuga* and *Fuscidea kochiana* , thrived under stabilized and nutrient‐enriched conditions. CCA showed that successional age explained 12.9% of the variability in species composition, whereas geographic differences contributed 7.9%. Species turnover was influenced by substrate characteristics (fine‐particle sediment vs. coarse rocky substrate), competition, and erosion, which was still shaped by environmental stability. Lichen succession follows the expected pattern, primarily shaped by terrain microtopography, climate, and substrate type. To refine the successional dynamics of lichen communities and other groups of organisms involved in the colonization of newly deglaciated habitats, more long‐term studies from different regions will be needed.

## Graphical abstract

We investigated the succession of lichens in glacier forefields in Sweden and Norway to assess how retreating ice shapes early lichen colonization and diversity patterns. Our results reveal consistent successional trends across sites and highlight the role of time since deglaciation and local environmental conditions in structuring lichen communities. These findings contribute to understanding biodiversity dynamics in newly exposed alpine habitats under climate change.

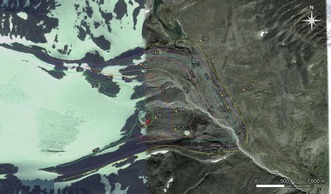

## 1 Introduction

Scandinavian glaciers defrosting has been carefully monitored for a long time, and stripped zones are ideal for studying succession of organisms. Storglaciären has been a key site for studying Holocene glacier dynamics in Swedish Lapland from the late 19th century to the 20th century (Karlén 1973). Intensive research is still ongoing here (Jansson 2008; Koblet et al. 2010). Numerous zoological succession studies have been conducted in the foreland of the Midtdalsbreen glacier over the past 25 years, providing valuable insights into the colonization dynamics of invertebrates in newly deglaciated habitats (Bråten et al. 2012; Flø and Hågvar 2013; Hågvar 2012; Hågvar and Gobbi 2022; Hågvar and Ohlson 2013; Hågvar et al. 2016; Klopsch et al. 2023). Research in the Finse area is extensive and long‐term (Østbye 1997). Several authors have focused on studying vegetation succession in deglaciated areas close to Finse (Berg 1976; Elven 1975; Elven and Ryvarden 1975a). The melting of glaciers has been monitored for a long time, and therefore, numerous studies from various continents focus on succession and landscape development after deglaciation. However, there are fewer studies tracking the succession of lichens as pioneer organisms in glacial forelands, specifically focusing on community development processes and the influence of environmental factors. Articles focused on higher plants or lichenometry provide data on how lichens establish, grow, and persist on recently exposed surfaces, contributing to a broader understanding of lichen and bryophyte succession in glacial environments (Karlén and Black 2002). Succession of various groups of organisms in deglaciated areas in Scandinavia and Svalbard is addressed by a wide range of studies (Haugland and Beatty 2005; Holt et al. 2006; Inoue et al. 2019; Koblet et al. 2010; Matthews and Vater 2015; Matthews and Whittaker 1987; Moreau et al. 2008; Phinney et al. 2022; Prach et al. 2012; Rachlewicz et al. 2007; Robbins and Matthews 2009; Rydgren et al. 2014; Sørlie 2001; Stork 1963; Vanneste et al. 2017; Vetaas 1994; Wietrzyk et al. 2018; Wietrzyk‐Pełka et al. 2018; Wietrzyk‐Pełka et al. 2021). Research in the European Alps (Bilovitz et al. 2015; Birks and Birks 2013; Erschbamer et al. 2023; Fickert 2020; Fischer et al. 2019; Nascimbene et al. 2017) identified a positive relationship between lichen species richness and time since deglaciation, highlighting mechanisms of directional species accumulation and trait selection. In Siberia, the succession of vascular plants, bryophytes, and lichens of deglaciated areas was studied by (Timoshok et al. 2020). Extensive research on succession is also being conducted in Antarctica and South America (Arróniz‐Crespo et al. 2014; Beck et al. 2019; Bohuslavová et al. 2018; Boy et al. 2016; Fernández‐Martínez et al. 2017; Frenot et al. 1995; Garrido‐Benavent et al. 2020; Llambí et al. 2021; Sancho et al. 2011; Vargas 2018) and in North America (Cooper 1923; Spribille et al. 2020). A list of sources used to create successional species scores, including the type of disturbance that initiated lichen succession, is summarized (Holt et al. 2006). Many detailed mechanisms and processes of succession and relations to environmental factors are available in (Frenot et al. 1995; Matthews and Vater 2015; Wietrzyk‐Pełka et al. 2021).

This research builds on the results of previous studies and focuses on site‐specific factors, spatial heterogeneity, and the role of pioneer species in Scandinavian glacial forefields. Studies from the Alps and Svalbard have described successional patterns (Bilovitz et al. 2015; Wietrzyk‐Pełka et al. 2021). Our research focused on local differences in colonization rates and species turnover due to microtopography, substrate type, and environmental conditions. By comparing chronosequences at two different glacier forefields (Storglaciären and Midtdalsbreen) and using detailed area‐based sampling and canonical correspondence analysis, we aimed to confirm the occurrence of pioneer species, such as *Stereocaulon* spp. and *Cetraria* spp. reported at different successional stages (Holt et al. 2006; Nascimbene et al. 2017). This research contributes to a more nuanced understanding of lichen succession in different glacial regions and refines predictions of species dynamics and community assembly in postglacial landscapes.

### 1.1 Aims of Our Study

1. Determine the time zones of succession of terricolous and saxicolous species after the glacier defrost. To look for common ecological properties among pioneer species and thallus type.
2. Divide the lichen species into time periods by their colonization rate.
3. Compare differences among dated plots of the Midtdalsbreen glacier foreland (Norway) and Storgläciaren (Sweden).

## 2 Materials and Methods

### 2.1 Study Sites

Storglaciären—The first of the studied deglaciated area is placed bellow the glacier Storglaciären (Swedish for the Grand Glacier), Kebnekaise Mt. in Tarfala valley in the Scandinavian Alps, Sweden (67.90235° N, 18.60596° E). Its front is at an altitude of about 1100 m above sea level (Stork 1963). The polythermal glacier have both cold and warm bottom temperatures. The main body of the Kebnekajse massif at Tarfala valley is composed from amphibolite alternating with micaschist and gneiss (Johansson 1951). The retreat of the Storglaciären glacier in the Kebnekaise massif in northern Sweden has been systematically documented through photography since the early 20th century (Stork 1963). Storglaciären has had a cumulative negative mass balance of −17 m between 1946 and 2006 (Jansson et al. 2008). The oldest (terminal) moraine begins approximately 800 m from the glacier margin. The topography of the ground moraine is strongly disturbed by rivers transporting meltwater. The marginal part of the moraine is located higher and is rich in siliceous boulders. A part ahead of the margin is located lower because of the fine material has been carried away by meltwater and rain. The outer part of the ground moraine area is very dark‐colored due to the presence of lobate and fruticose lichen thalli, which are dominant in areas without snow cover during winter time. The vegetation season take time from June to August with the mean temperature about 7°C–10°C (Bolin 2023). The succession of plants was detaily described by Stork (1963). In earliest successional stages up to 20 years after deglaciation dominate bryophytes and lichens ( *Psoroma hypnorum* and *Stereocaulon* sp.). In the latest successional stage saxicolous species *Umbilicaria* sp. appear.

Midtdalsbreen.—The second study area belongs to Midtdalsbreen glacier foreland (Hordaland, central Norway, 60.57389° N, 7.46920° E). It is a north‐eastern part of a huge glacier of Hardangerjøkulen, an ice cap on the northwestern border of the Hardangervidda plateau in southern Norway. A small valley glacier (6.8 km^2^) lies entirely above the tree line in the low‐ and mid‐alpine belts between 1100 and 1550 m (Matthews and Whittaker 1987). The glacier covers an elevation range from 1380 to 1865 m a.s.l. (Giesen, Andreassen, et al. 2009). Midtdalsbreen flows into the valley Finsedalen, which has an approximate west–east orientation. The vegetation season takes time from June to August, with the mean temperature about 10°C–12°C and a mean annual precipitation of 1000–1500 mm (CustomWeather 2023). Annual air temperature −1.2°C directly on the glacier (Giesen, Van den Broeke, et al. 2008). The bedrock of the Midtdalsbreen valley is composed of amphibolite alternating with both granite, gneiss, and phyllite (Reinardy et al. 2013). Midtdalsbreen has advanced 36 m between 1982 and 2001 and a cumulative negative mass balance of −219 m between 2001 and 2018 (Reinardy et al. 2019). The Midtdalsbreen glacier valley differs from the previous one by a more than 1 km long foreland (1200–1300 m) and a vast alpine region of 1300–1400 m a.s.l. The terminal moraine starts at about 1500 m distance from the ice margin. The topography below the moraine 2000 is strongly disturbed by rivers transporting meltwater. There is a significant rock degree. The boundaries of the oldest moraines (1750, 1930 and 1955) are well visible (see Figure 2). The succession of higher plants was detailed described by (Dahl 1984; Elven 1974; Elven and Ryvarden 1975b; Faegri 1967). The succession of vascular plants and ectomycorrhiza was studied in the deglaciated area by Sørlie (2001) and Vetaas (1994).

## 3 Sampling Design

**FIGURE 1:**
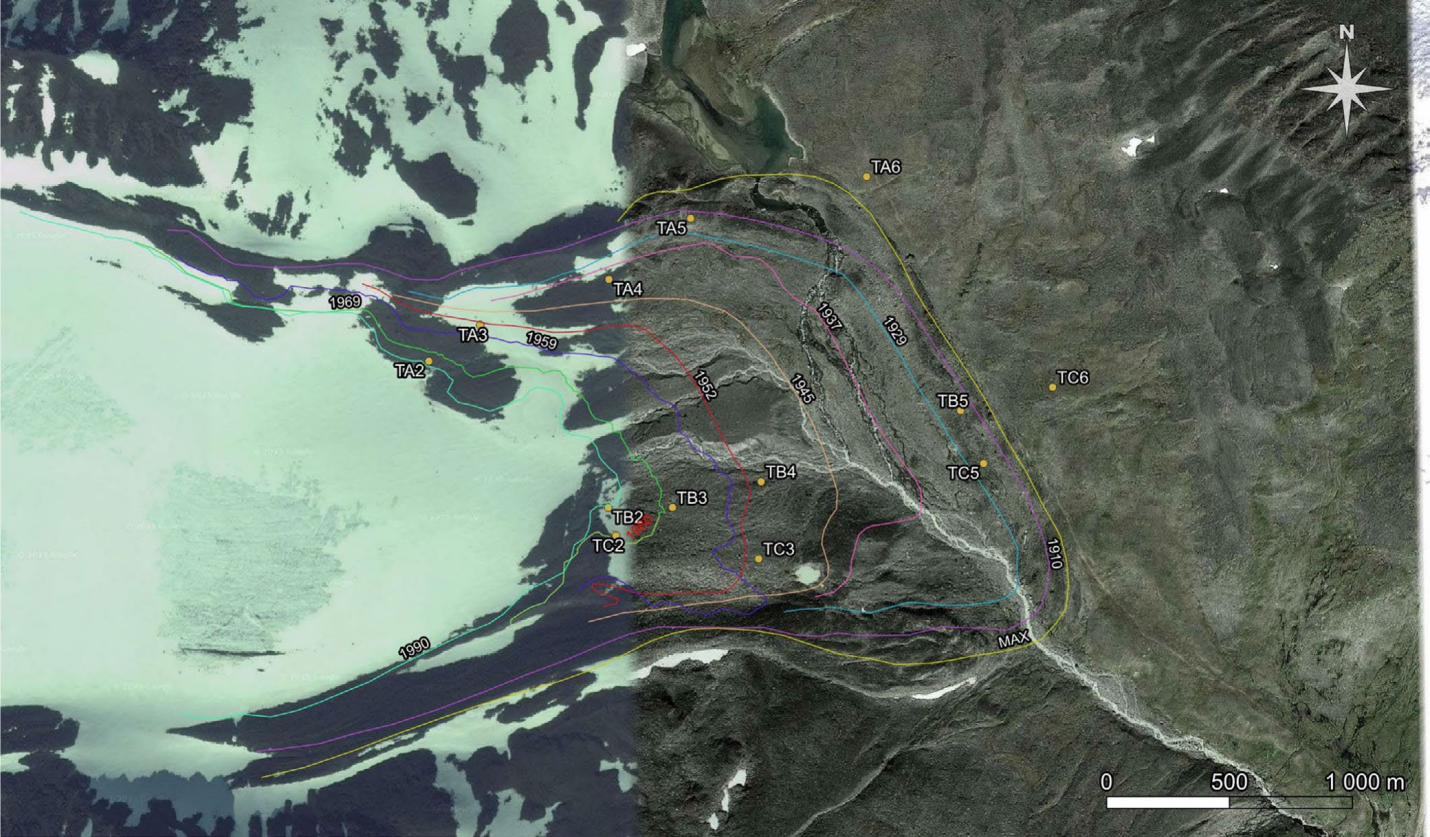
Location of the studied glacial forelands of Storglaciären (Sweden) and location of lineages and plots.

**FIGURE 2:**
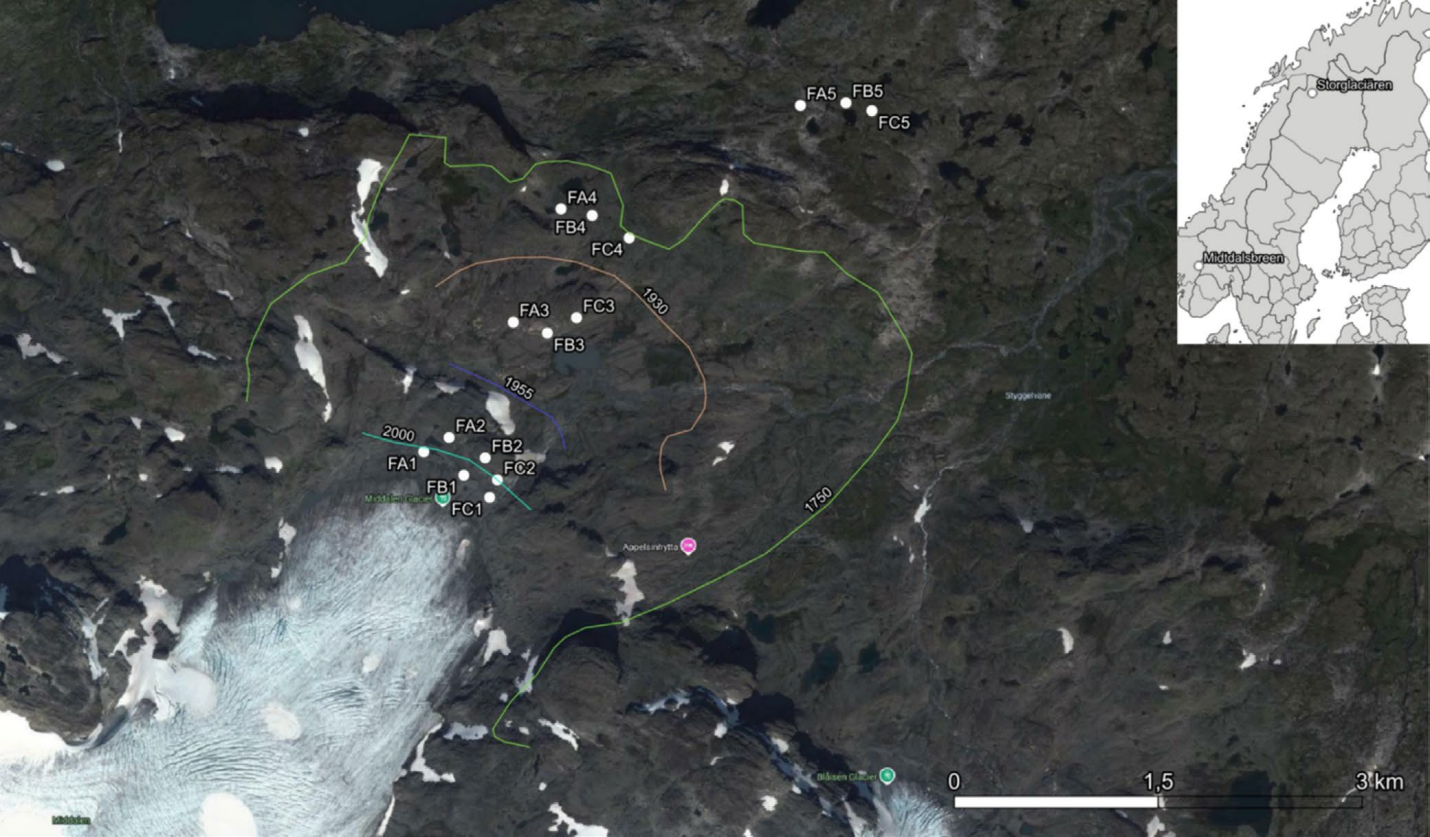
Location of the studied glacial forelands of Midtdalsbreen (Norway) and location of lineages and plots.

The monitoring of the plots was carried out during the 1st and 3rd weeks of August 2014. In each glacier foreland, five 5 m × 5 m plots (each with five subplots 1 m × 1 m, divided into 100 small squares of 10 × 10 cm, in which lichen species diversity and abundance were recorded in each cell. For the purposes of statistical analysis, the values were summed for each 5 × 5 m plot). 5 × 5 m plots with known years since deglaciation (Storglaciären: 24, 45, 62, 85, and 264 years—Figure 1; Midtdalsbreen: 9, 14, 59, 84, and 264 years—Figure 2) were randomly selected and positioned within three transects oriented W–E (Storglaciären) and S–NE (Midtdalsbreen) using a GIS application. Accurately measured lineages of retreating glaciers were used. We have shape files with well‐dated chronosequences of deflated areas from Tarfala and Finse research station. Transects are not linear because parts of the moraine are constantly disturbed by melting glacier water.

### 3.1 Zones

Five (Storglaciären—Figure 1, Table 1) and four (Midtdalsbreen—Figure 2) zones in each of the studied glacial forelands were delimited according to (Rachlewicz et al. 2007). The data set comprises 27 plots (135 subplots) from five terrain age classes (ca. 9–264 year after deglaciation). Data of the lichen species abundance (percentage cover) and a suite of relevant environmental variables (orientation, declination, ratio of stone and soil coverage) were recorded in each plot.

**TABLE 1:** Zones of the studied glacial forelands.

Storglaciären		Midtdalsbreen	
Ice free zone	Age	Ice free zone	Age
1990–2014	24	2005–2001	9
1969–1990	45	2000–2005	14
1952–1969	62	1955–2000	59
1929–1952	85	1930–1955	84
LIA	264	LIA	264

### 3.2 Data Analyses

All data supporting the findings of this study are available in https://doi.org/10.17632/mrpy38wbwb.1.

Cover (cm^2^) of each species in the plot 5 m × 5 m was expressed as the sum of the species percentage covers in the subplots 1 m × 1 m. Number of the subplots (nearly always 5) in the plot served as a weight of the plot (of the 27 plots, the lowest weight was thus assigned to one plot consisting of just one subplot and to two plots containing two subplots each). Although the plots in both localities were placed along several approximatelly linear transects (in order to cover all zones of deglaciation), there were no marks of obvious autocorrelation among plots within a transect. In particular, there was no reason to expect that species composition of the plots within a transect was more homogeneous due to existence of such linear vectors of lichen diaspores as water flows, avalanche pathways or wind currents that would follow directly the transects. We therefore treated the plots as independent observational units.

Because of considerable heterogeneity (3 SD‐units) in species composition of lichen communities in the plots, unimodal ordinations were used, according to (Šmilauer and Lepš 2014). Detrended correspondence analysis (DCA) was used for identification of main gradients in species composition.

Since the data were collected at two distant sites, the effect of geographic position on species composition of lichen communities was evaluated at first. This was performed by canonical correspondence analysis (CCA) with the locality coded as a factorial explanatory variable with two levels (Tarfala and Finse). Another CCA, with time after deglaciation as the only explanatory variable and locality as the factorial covariate, was then used to examine the effect of successional age (years after deglaciation) of a plot on the fraction of variability of species composition that could not have been explained by geographical distance of the study sites. To assess statistical significance of the relations between explanatory (locality or time after deglaciation) and response variables (species covers) Monte Carlo permutation tests with 499 unrestricted permutations were used.

Prior to the analyses, covers of lichen species and time after deglaciation were logarithmically transformed [*x*'= log (*x* + 1) and *x*' = log *x* for species covers and time after deglaciation, respectively]. The transformation reflects the suggestion of faster changes in species composition in early successional phases.

## 4 Results

### 4.1 Species Richness and Successional Phases

**FIGURE 3:**
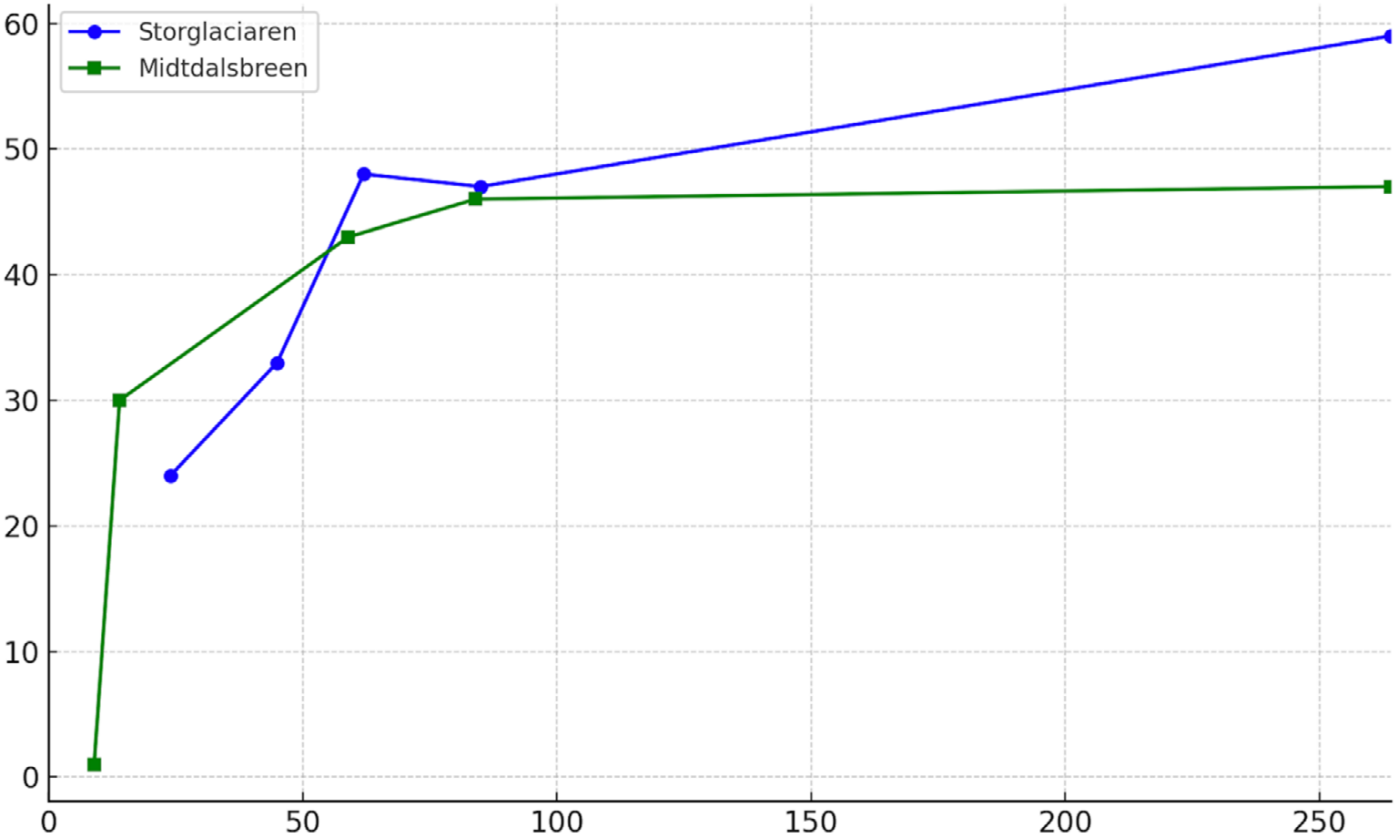
Species diversity comparisons of Storglaciären and Midtdalsbreen: *x*‐axis: Age (years), *y*‐axis: Number of species.

Lichen species richness increased rapidly in early successional stages (15–25 years post‐deglaciation) but stagnated or slightly decreased in older plots (> 100 years), suggesting competitive exclusion or resource limitation in late stages (Table 2, Figure 3).

**TABLE 2:** With average percentage cover (lichens on plots in different terrain age).

Storglaciären		Midtdalsbreen	
Ice free zone	Number of species	Ice free zone	Number of species
1990–2014	24	2005–2001	1
1969–1990	33	2000–2005	30
1952–1969s	48	1955–2000	43
1929–1952	47	1930–1955	46
LIA	59	LIA	47

### 4.2 Pioneer Species Identification

Early successional plots were dominated by pioneers, such as *Alectoria ochroleuca*, *Aspilidea myrinii*, *Stereocaulon alpinum*, and *Cetraria islandica* . These species played a critical role in initial substrate colonization, facilitating further biological succession.

### 4.3 Late‐Successional Species

Older plots (> 100 years) were characterized by species like *Arctoparmelia centrifuga*, *Fuscidea kochiana* , and *Nephroma arcticum* , indicating that these lichens thrive under stabilized and nutrient‐enriched conditions.

### 4.4 Significant Environmental and Geographic Influence

CCA revealed that both successional age and site‐specific geographic differences significantly influenced species composition. Geographic location explained 7.9% of variability, whereas successional age explained 12.9%.

### 4.5 Differences Between Storglaciären and Midtdalsbreen

Although both forelands shared common pioneer and late‐successional species, slight variations in species abundance and turnover rates highlight the role of localized environmental factors, such as microclimate and substrate characteristics.

### 4.6 Successional Trajectories and Turnover Patterns

The transition from pioneer to mid‐ and late‐successional species was gradual, with key turnover points driven by changes in substrate availability and competition. Species such as *Cetraria islandica* and *Flavocetraria nivalis* persisted throughout various stages, indicating broad ecological adaptability.

**FIGURE 4:**
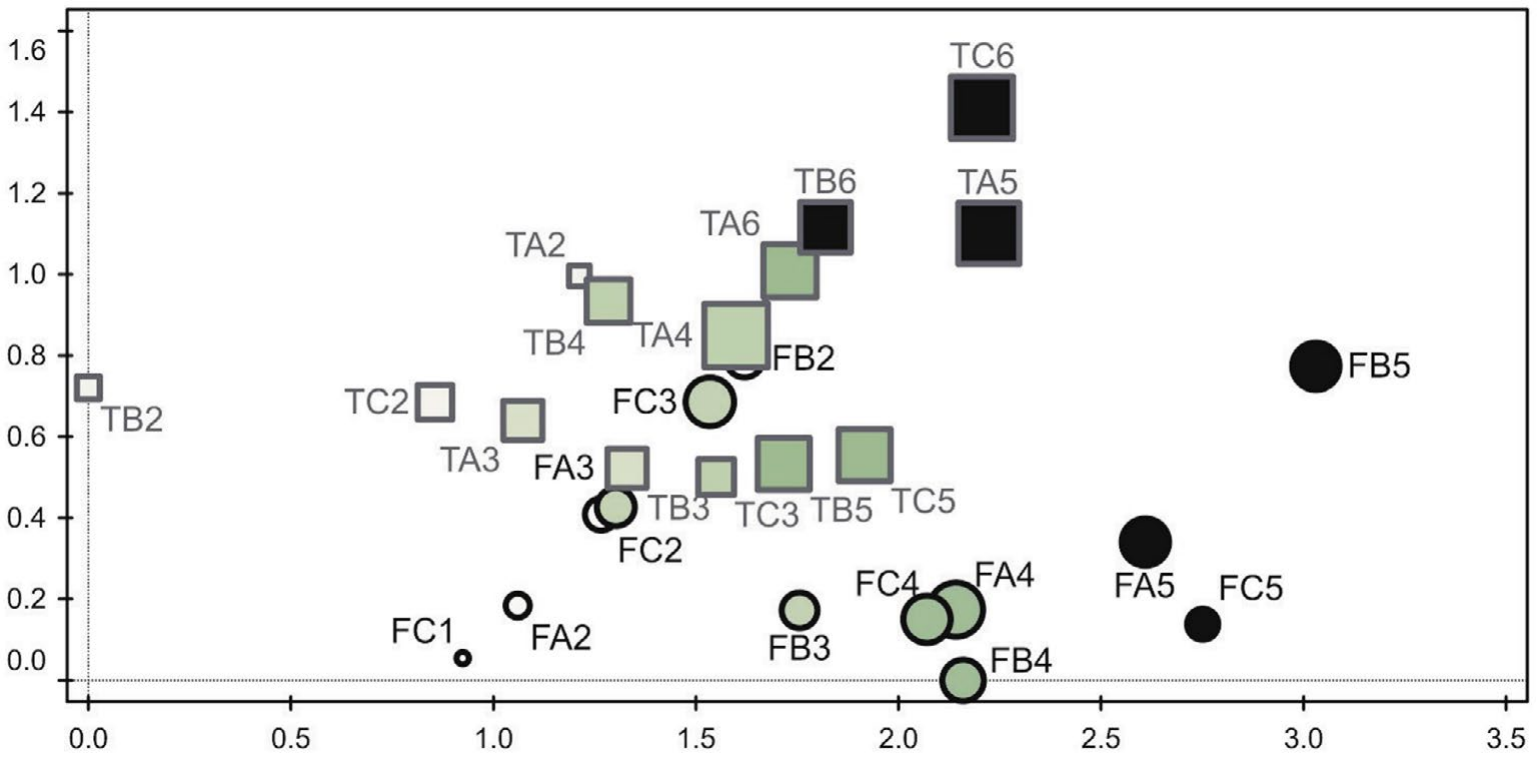
DCA ordination. Abbreviations of sampling plots in this figure: T squares = Tarfala, F circles = Finse. The size of the symbols corresponds to the number of species (the largest symbol = the highest species richness).

Ordination of the plots between the first two axes of the DCA suggested that the most important gradients of variability in species composition of lichen communities corresponded to geographic effects (differences in lichen biota between Tarfala and Finse) but also to successional age (time after deglaciation). The lowest species numbers were found in the youngest deglaciated plots. Species richness increased with time after deglaciation, culminated in intermediate successional stages, and stagnated or even decreased in the oldest deglaciated plots (Figure 4). Ordination of plots (5 m × 5 m) in DCA of lichen communities from deglaciated areas of various age at Storglaciären (Tarfala, T, squares) and Midtdalsbreen (Finse, F, circles). The first (horizontal) and the second (vertical) ordination axes explained 15% and 7%, respectively, of total variation in species composition of lichen communities. Size of symbols reflects the number of species in a plot. Color of symbols represents the gradient of successional age (time after deglaciation), from the youngest (white) through the intermediate (gray) to the oldest (black).

**FIGURE 5:**
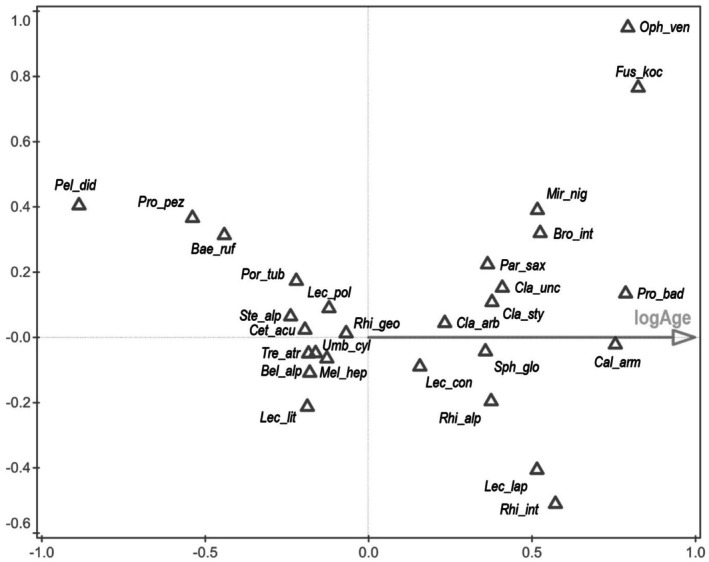
CCA ordination. Abbreviations of species in this figure: *Alectoria ochroleuca* (*Ale_och*), *Arctoparmelia centrifuga* (*Arc_cen*), *Baeomyces rufus* (*Bae_ruf*), *Bellemerea alpina* (*Bel_alp*), *Bilimbia lobulata* (*Bil_lob*), *Brodoa atrofusca* (*Bro_atr*), *Brodoa intestiniformis* (*Bro_int*), *Bryocaulon divergens* (*Bry_div*), *Caloplaca stillicidiorum* (*Cal_sti*), *Calvitimela armeniaca* (*Cal_arm*), *Cetraria aculeata* (*Cet_acu*), *Cetraria islandica* (*Cet_isl*), *Cladonia amaurocraea* (*Cla_ama*), *Cladonia arbuscula* (*Cla_arb*), *Cladonia bellidiflora* (*Cla_bel*), *Cladonia borealis* (*Cla_bor*), *Cladonia cervicornis* (*Cla_cer*), *Cladonia digitata* (*Cla_dig*), *Cladonia furcata* (*Cla_fur*), *Cladonia gracilis* (*Cla_gra*), *Cladonia macilenta* (*Cla_mac*), *Cladonia pleurota* (*Cla_ple*), *Cladonia pyxidata* (*Cla_pyx*), *Cladonia stygia* (*Cla_sty*), *Cladonia subulata* (*Cla_sub*), *Cladonia uncialis* (*Cla_unc*), *Epilichen scabrosus* (*Epi_sca*), *Flavocetraria cucullata* (*Fla_cuc*), *Flavocetraria nivalis* (*Fla_niv*), *Fuscidea kochiana* (*Fus_koc*), *Lecanora intricata* (*Lec_int*), *Lecanora polytropa* (*Lec_pol*), *Lecidea confluens* (*Lec_con*), *Lecidea fuscoatra* (*Lec_fus*), *Lecidea lapicida* (*Lec_lap*), *Lecidea lithophila* (*Lec_lit*), *Lecidea plana* (*Lec_pla*), *Lecidoma demissum* (*Lec_dem*), *Lepraria neglecta* (*Lep_neg*), *Melanelia hepatizon* (*Mel_hep*), *Melanelia stygia* (*Mel_sty*), *Micarea botryoides* (*Mic_bot*), *Micarea lignaria* (*Mic_lig*), *Miriquidica nigroleprosa* (*Mir_nig*), *Nephroma arcticum* (*Nep_arc*), *Ochrolechia parella* (*Och_par*), *Ochrolechia frigida* (*Och_fri*), *Ophioparma ventosa* (*Oph_ven*), *Parmelia saxatilis* (*Par_sax*), *Peltigera aphthosa* (*Pel_aph*), *Peltigera canina* (*Pel_can*), *Peltigera didactyla* (*Pel_did*), *Peltigera frippii* (*Pel_fri*), *Physcia dubia* (*Phy_dub*), *Placynthiella icmalea* (*Pla_icm*), *Placynthiella oligotropha* (*Pla_oli*), *Porpidia crustulata* (*Por_cru*), *Porpidia tuberculosa* (*Por_tub*), *Protopannaria pezizoides* (*Pro_pez*), *Protoparmelia badia* (*Pro_bad*), *Pseudephebe pubescens* (*Pse_pub*), *Pseudevernia furfuracea* (*Pse_fur*), *Psoroma hypnorum* (*Pso_hyp*), *Rhizocarpon alpicola* (*Rhi_alp*), *Rhizocarpon badioatrum* (*Rhi_bad*), *Rhizocarpon geographicum* (*Rhi_geo*), *Rhizocarpon intersitum* (*Rhi_int*), *Rhizocarpon norwegicum* (*Rhi_nor*), *Schaereria fuscocinerea* (*Sch_fus*), *Solorina crocea* (*Sol_cro*), *Sphaerophorus fragilis* (*Sph_fra*), *Sphaerophorus globosus* (*Sph_glo*), *Sporastatia testudinea* (*Spo_tes*), *Stereocaulon alpinum* (*Ste_alp*), *Thamnolia vermicularis* (*Tha_ver*), *Trapeliopsis granulosa* (*Tra_gra*), *Tremolecia atrata* (*Tre_atr*), *Umbilicaria cylindrica* (*Umb_cyl*), *Umbilicaria deusta* (*Umb_deu*), *Umbilicaria proboscidea* (*Umb_pro*), *Umbilicaria torrefacta* (*Umb_tor*).

CCA revealed a significant effect of locality on species composition of lichen communities (7.9% of explained variability, pseudo‐F = 2.1, *p* = 0.002). CCA, which took the influence of locality into account (i.e., with the locality as the covariate), found a significant effect of successional age (log‐transformed time after deglaciation) on species composition (12.9% of explained variability, pseudo‐F = 3.6, *p* = 0.002). The first (canonical) axis of the latter CCA represented the gradient of successional age. The lowest scores on the axis were achieved by the species that preferred newly deglaciated plots, e.g., *Alectoria nigricans*, *Aspilidea myrinii*, *Baeomyces rufus*, *Cetraria aculeata*, *Cetraria islandica* , *Cladonia* spp., *Flavocetraria cucullata*, *F. nivalis* , *Lecidea fuscoatra*, *L. lithophila*, *Lecanora polytropa* , *Lepraria neglecta agg*., *Melanelia hepatizon*, *M. stygia*, *Micarea botryoides*, *M. lignaria* , *Ochrolechia frigida* , *Peltigera didactyla* , *Porpidia tuberculosa*, *Pseudephebe pubescens* , *Psoroma hypnorum* , *Rhizocarpon badioatrum*, *Rhizocarpon norwegicum*, *Solorina crocea*, *Stereocaulon alpinum*, *Thamnolia vermicularis* , *Trapeliopsis granulosa* , *Tremolecia atrata*, and *Umbilicaria* spp. The highest scores, on the other hand, were reached by the species most abundant in the late successional phases, such as *Arctoparmelia centrifuga*, *Calvitimela armeniaca*, *Fuscidea kochiana* , *Nephroma arcticum* , *Ochrolechia parella* , *Protoparmelia badia* , *Ophioparma ventosa*, and *Sphaerophorus fragilis*; Table 1 and Figure 5.

Ordination of species in CCA of lichen communities from deglaciated areas of various age at Storglaciären (Tarfala) and Midtdalsbreen (Finse)—Figure 5. For clarity, only 28 species best fitted by the model are displayed. The first (horizontal) canonical axis represented the significant effect of successional age (time after deglaciation) on species compostion (12.9% of explained variability, pseudo‐F = 3.6, *p* = 0.002) after subtraction of the geographic effect (differences in lichen communities between Tarfala and Finse). The species with the lowest scores are usually found in early succession, whereas the species with the highest scores prefer the late successional phases after deglaciation.

### 4.7 Chronology of the Pioneer Zone

In total, 104 lichen species were found in the 27 sampling sites. The species composition differs slightly in the areas in Storglaciären (89) and Midtdalsbreen (81). The diversity of terricolous lichens near the front of the glacier was very low (one lichen species *Stereocaulon alpinum*). The first lichens occurred at a distance of about 80 m from the front (sites FA2, FB2, FC2, TA2, TB2 and TC2), and only 30 species were found in that sampling site.

The lichens *Stereocaulon alpinum*, *Cetraria islandica* , *and Flavocetraria nivalis* were the most frequent fruticose species (frequency of 106, 80 and 61) meanwhile, the foliose lichen *Solorina crocea* occurred with a frequency of 40. Among saxicolous lichens were frequent *Rhizocarpon geographicum* (93), *Umbilicaria proboscidea* (92), *Umbilicaria cylindrica* (81), *Lecanora polytropa* (78) and *Tremolecia atrata* (66). 51 species occurred with low frequency values (< 20).

## 5 Discussion

### 5.1 Complex Factors Driving Lichen Succession

Lichen succession is shaped by the combined effects of microclimate, substrate type, and disturbance regimes. Variability in these factors explains the differences observed between Storglaciären and Midtdalsbreen forelands. Similar conclusions were reached in a lichenological study on glaciers in the Alps (Nascimbene et al. 2017). In the youngest successional stages (approximately 25 years after deglaciation), an average of ~7 lichen species was recorded, whereas in the oldest stages (around 150 years), about 27 species were found. Among the first colonizers was *Stereocaulon alpinum* (Nascimbene et al. 2017). The main factor influencing lichen species diversity is the age of the substrate (time since deglaciation). A key factor for successful colonization is the type of photobiont, with cyanobacterial photobionts being significantly more successful in the early stages of succession. In some areas, bryophyte–cyanobacteria associations contribute to nitrogen accumulation very early after deglaciation, supporting soil development and preparing microhabitats for subsequent succession stages (Arróniz‐Crespo et al. 2014). In Svalbard, chlorolichens are initially better suited to extract water efficiently from high air humidity (Wietrzyk‐Pełka et al. 2018). *Stereocaulon* and *Cladonia* mention other works as first colonizers (Rydgren et al. 2014). Additional lichens with foliose ( *Peltigera rufescens* ) and fruticose ( *Cetraria islandica* ) thalli join the pioneer species, complementing the stands of *Stereocaulon alpinum* (Bilovitz et al. 2015; Erschbamer et al. 2023; Holt et al. 2006). (Fickert 2020), in his study, states that early successional vascular plant species do not disappear but are gradually supplemented by others. We observed the same pattern in our plots for certain lichen species. The rate of change in species composition is not linear. Primary succession proceeds rapidly at first and gradually slows down over time (Rydgren et al. 2014).

### 5.2 The Role of Microtopography in Primary Succession

Microtopography represents a significant but context‐dependent factor influencing succession on deglaciated terrain. Its impact is particularly evident during the early stages of succession, where fine‐scale relief structures affect the establishment and growth of pioneer species. However, the effectiveness of microrefugia is not universal and depends on their interaction with regional climatic conditions and the spatial scale of assessment. In a study from the foreland of the Sperry Glacier (USA) (Bryant et al. 2025) demonstrated that topographic features, such as depressions, drainage characteristics, and associated erosion exposing boulders significantly influence plant species diversity and vegetation cover. Microsites referred to as “safe sites” are most suitable for seed establishment and survival, and therefore critically shape the direction and speed of succession. Microtopography also affects energy availability (e.g., solar radiation depending on slope exposure) and snow retention, thus modulating the onset of succession (Bayle et al. 2023). Differences in substrate particle size, slope steepness, and depressions determine the divergence of succession trajectories. Fine‐grained sediments on convex landforms support the development of grasses, whereas concave shapes with long‐lasting snow cover favor the genera *Salix* and *Carex* (Dolezal et al. 2008). According to (Chytrý et al. 2024), the role of microrefugia may be overestimated if the spatial dynamics of species dispersal (so‐called spatial mass effects) are not taken into account. They showed that many plant species and community attributes are better predicted by topographic variables at scales above 20 m than by microtopographic features at 1–5 m. Therefore, spatial effects (e.g., species dispersal) can outweigh local microclimatic filters. (Malanson et al. 2024) highlight the regionally variable effect of microtopography depending on climatic regime. In humid regions (e.g., Glacier National Park), factors related to water retention are most influential, whereas in dry regions (e.g., New Mexico), radiation and temperature variables dominate. From the perspective of conserving biodiversity, microtopography acts as a climatic filter, whose effectiveness is determined by a complex suite of environmental variables. For the survival of cold‐adapted species, accumulations of cold air in topographic depressions—so‐called cold‐air pooling—can be significant (Pastore et al. 2022). These microclimatic pockets may play a crucial role in mitigating the impacts of global warming.

### 5.3 Pioneer Species Establishment

Pioneer species such as *Stereocaulon alpinum* and *Cetraria islandica* are adapted to extreme conditions (drought in the form of frost), allowing their small thallus fragments to easily establish on sheltered terrain irregularities (Bohuslavová et al. 2018; Nascimbene et al. 2017; Rydgren et al. 2014). With increasing lichen abundance, the microclimate and surface of the microsite change. Water retention capacity improves, creating favorable conditions for the establishment of other organisms (Holt et al. 2006). Lichen colonization is highly influenced by substrate stability. Water or wind erosion significantly slows succession. Pioneer species establish on the substrate only once it is sufficiently stable, sometimes several decades after ice retreat (Bilovitz et al. 2015; Nascimbene et al. 2017).

A study from the Alps lists mosses as the most effective colonizers in all areas, together with the lichens *Stereocaulon alpinum* and *Peltigera rufescens* , whereas in the later successional stage, lichens play a minor role compared to vascular plants, with only resilient species like *Stereocaulon alpinum* persisting as dominance shifts toward grasses and herbaceous plants, limiting lichen expansion (Erschbamer et al. 2023). The thalli of these lichens are long‐lived and capable of surviving for decades, forming a stable foundation for the developing community.

(Bilovitz et al. 2015) confirm similar patterns. *Peltigera rufescens* was identified as a pioneer species on surfaces up to 30 years old, whereas in older successional stages, *Cladonia pyxidata* , *Cladonia symphycarpia*, and again *Stereocaulon alpinum* were dominant. Another Alpine study (Fischer et al. 2019) describes a general successional pattern, where *Stereocaulon alpinum* dominates in the early stages (< 10 years), species of *Cladonia* occur during mid‐succession (10–25 years), and *Cetraria islandica* and *Alectoria ochroleuca* appear later (> 55 years).

In our plots, we observed a notable presence of *Protopannaria pezizoides* on fine sedimented material. This lichen, like the aforementioned species, contains a cyanobacterial photobiont capable of efficient nitrogen fixation. The decomposition of lichen biomass further creates a substrate for the growth of other plants—for example, we recorded mossy shrubs of the genus *Racomitrium*.

From the foreland of a Venezuelan glacier, pioneer species have been reported to include lichens *Cladonia* spp., *Rhizocarpon umbilicatum*, and bryophytes *Racomitrium* spp.; after 21 years, *Tremolecia atrata* appears (Llambí et al. 2021). In Chile, the first colonizers were lichens *Placopsis* spp., *Rhizocarpon geographicum*, *Xanthoria candelaria* , and *bryophytes Dendroligotrichum squamosum*, *Andreaea* spp., and *Racomitrium* spp. (Arróniz‐Crespo et al. 2014). Another Chilean study (Fernández‐Martínez et al. 2017) shows that in the pioneer stage (< 10 years), the dominant species included *Stereocaulon* sp., *Placopsis pycnotheca*, and mosses *Ditrichium cylindricarpum* and *Acroschisma wilsonii* , whereas older surfaces rapidly became overgrown with vascular plants.

At the same time, some previously unclear interpretations have been clarified—for example, *Stereocaulon alpinum* was considered a climax species in some studies (Moreau et al. 2008), but more recent research (including ours and that of (Wietrzyk‐Pełka et al. 2018)) confirms its role as an early colonizer of recently deglaciated habitats.

### 5.4 Lichen Succession Across Geographical Regions: Patterns and Dynamics

Across all regions, early successional stages tend to show significant variation in species composition between sites due to localized conditions and regional species pools. However, late successional stages tend to converge toward more uniform climax tundra communities. Although each forefield starts with a unique set of pioneer species, over time, the vegetation across different glacier forelands becomes more similar as climax species colonize from the regional pool. Our results from both Norwegian and Swedish glacier forelands support these patterns.

Similarly to previous studies (Holt et al. 2006), we observed the remarkable persistence of species, such as *Stereocaulon alpinum*, *Cetraria islandica* , and *Cladonia* spp. These species remain part of the community from the onset of succession for several decades and continue to persist until they are eventually outcompeted by shrubby angiosperms (e.g., *Calluna vulgaris* ). Early colonizers decline over time due to competition for light, space, water, and nutrients, as well as environmental changes, such as soil development, increased shading, and vegetation encroachment. This shift allows species like *Arctoparmelia centrifuga* and *Fuscidea kochiana* to thrive in later successional stages. Species turnover and competitive dynamics differ under various geographical conditions, influencing both the pace and direction of succession.

In regions with milder climates, such as southern Norway and the Alps, the succession of lichens and bryophytes proceeds relatively rapidly. According to (Haugland and Beatty 2005), the first lichens and mosses appear approximately 30 years after deglaciation, followed by the establishment of grasses and sedges. Woody shrub species become established only in the oldest areas. In these environments, a succession period of 150–250 years can be sufficient for the development of shrub‐dominated communities, including heathlands with climax indicators, such as *Vaccinium* spp. and *Betula nana* . These findings suggest that while the communities have not yet reached a true climax stage, they are well along the successional trajectory.

In contrast, on Svalbard, harsh climatic conditions slow down the successional process significantly. Frost erosion and limited growing seasons inhibit the development of vegetation. Bryophytes are the first to establish, followed by vascular plants, whereas lichens appear only in the later stages of succession (Wietrzyk et al. 2018). Even after 100 years, the vegetation corresponds more closely to a polar desert than to the climax tundra found in the surrounding landscape. The late‐successional stage is still dominated by lichen communities and a few hardy angiosperms (e.g., *Minuartia*), whereas shrubs or dwarf trees are absent (Wietrzyk‐Pełka et al. 2018). Lichen diversity on Svalbard varied between 24 and 82 species across different sites, with major differences attributed to substrate type, such as calcium‐rich rocks (Wietrzyk‐Pełka et al. 2018).

In the Altai, contrasting environmental conditions between neighboring glaciers can result in a time difference of several decades in the onset of rapid succession. (Timoshok et al. 2020) observed variation in successional timing driven by factors, such as microclimate, topography, and local disturbance.

In high‐altitude tropical ecosystems, such as the Andes, lichens and bryophytes also function as pioneering photosynthetic organisms (Llambí et al. 2021), playing a key role in early soil stabilization and nutrient cycling.

The chemical composition of the substrate is a critical factor influencing the rate of succession. For instance, limestone bedrock accelerates soil development and thus facilitates faster vegetation establishment (Wietrzyk‐Pełka et al. 2018).

### 5.5 Geographic and Temporal Variability

Within the same region, nearby glacier forefields can differ significantly. In Norway, generally similar successional patterns were observed across three glacier forefields, but community development varied locally due to the presence of microtopographic factors (e.g., rugged terrain vs. stable soil) (Haugland and Beatty 2005). Significant differences in lichen composition and richness were observed across eight glacier forefields on Svalbard. Local conditions (e.g., surface age after deglaciation, moisture, exposure) can lead to distinct successional outcomes (Wietrzyk‐Pełka et al. 2018). Successional dynamics are strongly shaped by geography, whether through broader climatic regions or local environmental factors (Wietrzyk‐Pełka et al. 2021).

The rate and course of succession vary between locations. (Matthews and Vater 2015) focusing primarily on arthropods, identified four successional stages over the first ~33 years at the Storbreen forefield in Norway, highlighting that even faunal succession can differ significantly across sites. Unstable phase (~0–6 years after deglaciation)—deposited sediments with no vegetation. Active stone surface phase (~6–21 years)—permeable substrate, colonization by herbs and bryophytes. Short transitional phase (~21–26 years)—gradual vegetation development. Rapid expansion phase (~26–33 years)—a sharp increase in vegetation cover. (Haugland and Beatty 2005) observed a similar development but did not specify the exact timing. In the Arctic, succession proceeds more slowly than in milder regions. (Wietrzyk et al. 2018) reported bryophytes as the first colonizers on Svalbard, later accompanied by pioneer vascular plants. On substrates several hundred years old, soil lichens undergo significant expansion. The species turnover timeframe is considerably extended in the cold and dry Arctic conditions compared to milder alpine forefields, where succession occurs more rapidly. Substrate stability and moisture play a key role in colonization development. Waterlogged and unstable soils hinder the colonization of angiosperms until a permeable substrate forms through water seepage and frost weathering over approximately 6 years (Matthews and Vater 2015). In the Maritime Antarctic, specifically on King George Island, clear successional changes in lichen and bryophyte communities have been observed on glacier forelands. During the early successional stages (0–20 years post‐deglaciation), pioneer species, such as the lichens *Cladonia pyxidata* , *Stereocaulon alpinum*, *Rhizocarpon geographicum*, and *Ochrolechia frigida* , along with bryophytes like *Polytrichum piliferum* , *Andreaea* sp., and *Sanionia uncinata* , establish on rocky surfaces and nutrient‐poor soils, often affected by cryoturbation. In mid‐successional stages (20–100 years), species diversity increases with the addition of taxa typical for more stabilized substrates, including *Cladonia symphycarpia*, *Cetraria islandica* , and *Flavocetraria nivalis*, accompanied by bryophytes, such as *Bryum amblyodon* and *Chorisodontium* sp. In late successional and climax communities (> 100 years), indicator species of stable vegetation appear, such as *Usnea fasciata*, *Xanthoria elegans* , and *Himantormia lugubris*, whereas some early colonizers like *Polytrichum piliferum* and *Sanionia uncinata* persist, indicating their long‐term survival potential (Boy et al. 2016).

These findings demonstrate that directional succession of cryptogam communities occurs even under the extreme conditions of Antarctica, mirroring patterns observed in other glacier forelands. They also highlight that certain species can persist across successional stages, whereas others serve as indicators of advanced vegetation development. This underscores the ecological importance of lichens and bryophytes not only as pioneers but also as key components of mature stages in primary succession.

### 5.6 Succession of Lichens on Other Reflected Sites

According to Holt's list of successional rates (Holt et al. 2006), the species composition during the early stages of succession (0–1 successional score on a scale of 1–3) is notably diverse. More than four studies from Holt's list identify the fastest colonizers (with a successional score of 1.5–1.8) as *Cladonia cornuta* , *Cladonia gracilis* , *Cladonia coccifera* agg., *Cladonia deformis* , *Cladonia crispata* , and *Stereocaulon paschale*. In the later stages of succession (scores of 2–3), species, such as *Cetraria islandica* , *Cladonia arbuscula* agg., *Cladonia uncialis* agg., *Cladonia rangiferina*, *Cladonia stellaris*, and *Flavocetraria nivalis* become more prevalent. In the Alps, *Peltigera rufescens* is reported as the first colonist on deglaciated surfaces (Bilovitz et al. 2015), whereas in Antarctica, species of *Usnea* sp. dominate the early stages (Bohuslavová et al. 2018; Boy et al. 2016). In the Alps, late‐stage colonizers include *Cladonia pyxidata* agg., *Cladonia symphycarpia*, and *Stereocaulon alpinum* (Bilovitz et al. 2015; Wietrzyk et al. 2018). Observed several pioneer basiphilous lichens on Svalbard, such as *Rostania ceranisca* and *Polyblastia cupularis*. *Stereocaulon alpinum* was found in both early and late successional stages. The occurrence of certain species is influenced by the availability of specific substrates, such as plant debris, which supports species like *Cladonia pyxidata* , *Lecidea epibryon*, *Lecidea ramulosa*, and *Ochrolechia androgyna* . Pioneer indicator species identified include *Atla wheldonii*, *Lathagrium cristatum*, *Polyblastia sendtneri*, and *Rostania ceranisca* (Wietrzyk‐Pełka et al. 2018).

According to (Moreau et al. 2008), *Stereocaulon alpinum* serves as an indicator of climax vegetation in the foreland of Midtre Lovénbreen, Austre Lovénbreen, and Midtdalsbreen. (Wietrzyk et al. 2018), however, do not classify *Stereocaulon alpinum* as a climax species and report its occurrence near the glacier front. In our study, *Stereocaulon alpinum* was often found in younger areas near the Storglaciären face. In our findings, the following species were identified as pioneers colonizing newly deglaciated substrates 15–25 years after glacier retreat: *Alectoria nigricans*, *Aspilidea myrinii*, *Baeomyces rufus*, *Cetraria aculeata*, *Cetraria islandica* , *Cladonia* spp., *Flavocetraria cucullata*, *Flavocetraria nivalis*, *Lecidea fuscoatra*, *Lecidea lithophila*, *Lecanora polytropa* , *Lepraria neglecta agg*., *Melanelia hepatizon*, *Melanelia stygia*, *Micarea botryoides*, *Micarea lignaria*, *Ochrolechia frigida* , *Peltigera didactyla* , *Porpidia tuberculosa*, *Protopannaria pezizoides*, *Pseudephebe pubescens* , *Rhizocarpon badioatrum*, *Rhizocarpon norwegicum*, *Solorina crocea*, *Stereocaulon alpinum*, *Thamnolia vermicularis* , *Trapeliopsis granulosa* , *Tremolecia atrata*, and *Umbilicaria* spp.

### 5.7 Lichen Succession After Fire Disturbance

Lichen succession in fire‐damaged areas shares similarities with post‐glacial colonization, as fires often reset successional trajectories and alter community structure. However, fire‐specific factors–such as intensity, frequency, and substrate type–distinctly shape recovery.

High‐intensity fires may eliminate the entire lichen layer, delaying recolonization and favoring pioneer species like *Cladonia pyxidata* , *Lecidea ramulosa*, and *Lecanora epibryon* on nutrient‐poor substrates (Moreau et al. 2008; Wietrzyk et al. 2018). Postfire microclimatic conditions (temperature, humidity, light) also influence lichen establishment. Some species, such as *Stereocaulon alpinum*, exhibit high adaptability, thriving in both early and late stages (Wietrzyk‐Pełka et al. 2018).

Recovery rates vary across regions. In subalpine Canada, *Cladonia* pioneers like *C. cristatella* appear within 7–10 years (Girard et al. 2017). In the West Siberian tundra, fruticose lichens ( *C. stellaris* , *C. rangiferina*) may take over 40 years to regain prefire coverage (Heim et al. 2021).

On the Kola Peninsula (Russia), *Trapeliopsis granulosa* appeared 8 years postfire, followed by *Cladonia* spp. after 15 years, *C. stellaris* and *C. rangiferina* after 80 years, and later *Nephroma arcticum* and *Peltigera aphthosa* after 150–200 years (Stavrova et al. 2020).

Mid‐ to late‐successional species, such as *Cetraria islandica* , *Flavocetraria nivalis*, and *Cladonia rangiferina* dominate later stages, contributing to organic matter accumulation and soil development, which facilitates vascular plant establishment (Bilovitz et al. 2015). In boreal forests, full recovery to climax communities spans decades, with frequent fires favoring pioneer dominance and delaying late‐successional colonizers like *Stereocaulon alpinum* and *Cladonia arbuscula* (Wietrzyk et al. 2018).

### 5.8 Climate Change and Lichen Communities

Lichens are sensitive to microclimatic changes caused by climate warming, which brings higher temperatures, more nutrients, and cloud cover in alpine ecosystems (Ndah et al. 2024). This change favors shrubs, such as heather and bilberry. Global tundra warming experiments (Elmendorf et al. 2012) show a decline in lichen cover of nearly 20% in 20 years and up to 50% for some species (Herk et al. 2002). There is a decline of terricolous shrub lichens on exposed tundra soils as well as rock species, with boreomontane species disappearing more slowly (Hauck 2009). In the Harz Mountains, species such as *Umbilicaria proboscidea*, *Catolechia wahlenbergii*, and others became extinct toward the end of the 20th century. Warming has also had a negative impact on, for example, *Flavocetraria cucullata* (Alatalo et al. 2017). The ability of lichens to migrate to new areas is limited (Mallen‐Cooper et al. 2023).

Climate change (increase in temperature, extension of the growing season, and more frequent and prolonged drought periods during the growing season) is a key factor in current primary succession processes. It slows down succession and completely alters natural successional processes (Losapio et al. 2018). Temperature is not always a direct cause of vegetational changes, but it affects other significant environmental factors: the frequency of frosts, permafrost development, continentality, fire frequency, and the environmental impact of glaciers (Birks and Birks 2013).

Climate warming is altering alpine ecosystem structure, favoring vascular plants and bryophytes over lichens (Vanneste et al. 2017). Until recently, pioneer communities in the Arctic faced little competition, as the development of fast‐growing species was limited by climatic factors. Warming and a longer growing season will enable the more aggressive expansion of vascular plants into glacier forefields. Photophilic lichens will be pushed to glacier margins or may disappear entirely (Wietrzyk‐Pełka et al. 2018). Warming will accelerate the establishment of late‐successional species and shorten the dominance period of pioneer species.

In regions with a milder climate, where the transition from the pioneer stage to climax communities occurs more rapidly, warming will further accelerate shrub expansion, leading to the displacement of natural vegetation (Birks and Birks 2013). The transition to shrub vegetation can be very rapid, occurring within a time frame of several decades (Fernández‐Martínez et al. 2017).

With accelerating glacier retreat due to climate change, shifts in successional trajectories and changes in species composition are likely, potentially impacting long‐term ecosystem stability (Arróniz‐Crespo et al. 2014; Llambí et al. 2021).

The species diversity and abundance of lichens are influenced by a complex interplay of environmental factors as climatic conditions, substrate type (soil quality, mineral bedrock) and erosion. Therefore, the species composition is different in some studies, and it is difficult to compare results. The results of our research did not stratify clear species corresponding to precisely dated periods. Species composition is influenced in different stages by several factors, and we managed to capture only some of them. The most important factors for the success of the species are a combination of morphological adaptation and environmental conditions like dominant species around, source of vegetative propagules (occurence of soredia or fragile thallus) and the structure of the terrain surface where particles can sediment. In order to generalize the results across different geographical areas and to assess the global implications of changing successional dynamics, the study highlights the need for long‐term, multiregional studies of lichen succession.

## 6 Conclusions

A comparative analysis of lichen succession in Storglaciären (Sweden) and Midtdalsbreen (Norway) confirms the fundamental role of environmental factors in community formation and successional dynamics. Primary succession follows a predictable trajectory, with rates and outcomes highly dependent on site‐specific conditions, such as substrate, microclimate, terrain character, and the influence of disturbance.

### 6.1 Species Diversity Trends

Lichen species richness increases rapidly during early successional phases (15–25 years post‐deglaciation) as pioneer species colonize newly exposed substrates.

The diversity stabilizes or slightly decreases in older plots (> 100 years), likely due to resource competition and environmental constraints.

### 6.2 Pioneer and Late‐Successional Species

Early colonizers such as *Stereocaulon alpinum* and *Cetraria islandica* dominate initial stages, stabilizing the surface and enabling later ecological development.

Late successional species, including *Arctoparmelia centrifuga* and *Fuscidea kochiana* , thrive under stable and nutrient‐enriched conditions.

### 6.3 Environmental Influence

CCA reveals that successional age explains 12.9% of the variability in species composition, whereas geographic factors (site‐specific differences) account for 7.9%. Variations in microtopography, substrate type, and microclimate between the two regions affect species turnover rates.

### 6.4 Comparative Differences

Although both regions share common pioneer and late‐successional species, Storglaciären shows slightly slower species turnover due to environmental constraints like microclimatic variability and different substrate characteristics compared to Midtdalsbreen.

### 6.5 Implications for Climate Change

Lichen succession is a very complex process. More long‐term studies from different geographical areas will be needed to refine succession models. Climate change accelerating glacier retreat is rapidly altering known successional processes. Understanding species and community turnover is essential for predicting future ecosystem change. Further research should focus on climate‐induced dispersal mechanisms and interactions between lichens, bryophytes, and vascular plants.

## Author Contributions

**Josef P. Halda:** conceptualization (equal), data curation (equal), investigation (equal), methodology (equal), project administration (equal), supervision (equal), validation (equal), visualization (equal), writing – original draft (equal). **Jan Košnar:** conceptualization (equal), data curation (equal), formal analysis (equal), methodology (equal), software (equal), supervision (equal), validation (equal), writing – original draft (equal). **Alena Lukešová:** conceptualization (equal), project administration (equal), supervision (equal).

## Conflicts of Interest

The authors declare no conflicts of interest.

## Data Availability

The data are openly available at https://doi.org/10.17632/mrpy38wbwb.1 and relevant.

## References

[ece371848-bib-0001] Alatalo, J. M. , A. K. Jägerbrand , S. Chen , and U. Molau . 2017. “Responses of Lichen Communities to 18years of Natural and Experimental Warming.” Annals of Botany 120: 159–170. 28651333 10.1093/aob/mcx053 PMC5737088

[ece371848-bib-0002] Arróniz‐Crespo, M. , S. Perez‐Órtega , l. R. A. De , et al. 2014. “Bryophyte‐Cyanobacteria Associations During Primary Succession in Recently Deglaciated Areas of Tierra del Fuego (Chile).” PLoS One 9, no. 5: e96081. 10.1371/journal.pone.0096081. 24819926 PMC4018330

[ece371848-bib-0003] Bayle, A. , B. Z. Carlson , A. Zimmer , et al. 2023. “Local Environmental Context Drives Heterogeneity of Early Succession Dynamics in Alpine Glacier Forefields.” Biogeosciences 20, no. 8: 1649–1669. 10.5194/bg-20-1649-2023.

[ece371848-bib-0004] Beck, A. , J. Bechteler , A. Casanova‐Katny , and I. Dzhilyanova . 2019. “The Pioneer Lichen Placopsis in Maritime Antarctica: Genetic Diversity of Their Mycobionts and Green Algal Symbionts, and Their Correlation With Deglaciation Time.” Symbiosis 79: 1–24. 10.1007/s13199-019-00624-4.

[ece371848-bib-0005] Berg, R. Y. 1976. “Etableringsprosjektet ‐ Etablering Av Primærproduksjon På Bar Fjellmark.” In IBP i Norge. Årsrapport 1974. Sluttrapport, edited by R. E. Vik . IBP i Norge.

[ece371848-bib-0006] Bilovitz, P. O. , A. Wallner , V. Tutzer , J. Nascimbene , and H. Mayrhofer . 2015. “Terricolous Lichens in the Glacier Forefield of the Pasterze (Eastern Alps, Carinthia, Austria).” Phyton 55, no. 2: 201–214. 10.12905/0380.phyton55(2)2015-0201. 26877565 PMC4746754

[ece371848-bib-0007] Birks, H. , and H. Birks . 2013. “Vegetation Responses to Late‐Glacial Climate Changes in Western Norway.” Preslia‐Praha 85: 215–237.

[ece371848-bib-0008] Bohuslavová, O. , P. Macek , O. Redčenko , K. Láska , L. Nedbalová , and J. Elster . 2018. “Dispersal of Lichens Along a Successional Gradient After Deglaciation of Volcanic Mesas on Northern James Ross Island, Antarctic Peninsula.” Polar Biology 41: 2221–2232. 10.1007/s00300-018-2357-7.

[ece371848-bib-0009] Bolin . 2023. “Monthly Mean Air Temperature at Tarfala 1946–2012.” https://bolin.su.se/data/tarfala/data/climate/tarfala_monthly_mean_temperature.txt.

[ece371848-bib-0010] Boy, J. , R. Godoy , O. Shibistova , et al. 2016. “Successional Patterns Along Soil Development Gradients Formed by Glacier Retreat in the Maritime Antarctic, King George Island.” Revista Chilena de Historia Natural 89, no. 6: 17. 10.1186/s40693-016-0056-8.

[ece371848-bib-0011] Bråten, A. T. , F. Daniel , H. Sigmund , H. Oddvar , M. C. E , and K. Aakra . 2012. “Primary Succession of Surface Active Beetles and Spiders in an Alpine Glacier Foreland, Central South Norway.” Arctic, Antarctic, and Alpine Research 44, no. 1: 2–15. 10.1657/1938-4246-44.1.2.

[ece371848-bib-0012] Bryant, A. , L. M. Resler , D. Gielstra , and T. Pingel . 2025. “Vegetation Succession Patterns at Sperry Glacier's Foreland, Glacier National Park, MT, USA.” Land 14, no. 2: 306. https://www.mdpi.com/2073‐445X/14/2/306.

[ece371848-bib-0013] Chytrý, K. , N. Helm , K. Hülber , et al. 2024. “Limited Impact of Microtopography on Alpine Plant Distribution.” Ecography 2024, no. 2: e06744. 10.1111/ecog.06744.

[ece371848-bib-0014] Cooper, W. S. 1923. “The Recent Ecological History of Glacier Bay, Alaska: The Present Vegetation Cycle.” Ecology 4, no. 3: 223–246. 10.2307/1929047.

[ece371848-bib-0015] CustomWeather . 2023. “Annual Weather Averages in Finse. Based on Weather Reports Collected During 2005–2015.” https://www.timeanddate.com/weather/norway/finse/climate.

[ece371848-bib-0016] Dahl, E. 1984. “En Oversikt Over Plantesamfunn På Finse.” Rapporter fra Høyfjellsøkologisk forskningsstasjon, Finse, Norge 1: 1–30.

[ece371848-bib-0017] Dolezal, J. , H. Kosuke , T. Koichi , Y. Valentine , and T. Hara . 2008. “Primary Succession Following Deglaciation at Koryto Glacier Valley, Kamchatka.” Arctic, Antarctic, and Alpine Research 40, no. 2: 309–322. 10.1657/1523-0430(06-123)[DOLEZAL]2.0.CO;2.

[ece371848-bib-0018] Elmendorf, S. C. , G. H. Henry , R. D. Hollister , et al. 2012. “Global Assessment of Experimental Climate Warming on Tundra Vegetation: Heterogeneity Over Space and Time.” Ecology Letters 15: 164–175. 22136670 10.1111/j.1461-0248.2011.01716.x

[ece371848-bib-0019] Elven, R. 1974. “Plant Communities on Recently Deglaciated Moraines at Finse, Southem Norway.” In IBP in Norway. Annual Report 1974, Appendix 1, edited by R. Vik , 381–467. Norwegian National IBP Committee.

[ece371848-bib-0020] Elven, R. 1975. “Plant Communities on Recently Deglaciated Moraines at Finse, Southern Norway.” In IBP in Norway. Methods and Results. Section PT‐UM Grazing Project, Hardangervidda. Botanical Investigations, edited by R. E. Vik . Norwegian National IBP Committee.

[ece371848-bib-0021] Elven, R. , and L. Ryvarden . 1975a. “Dispersal and Primary Establishment of Vegetation.” Ecological Studies 16: 82–85.

[ece371848-bib-0022] Elven, R. , and L. Ryvarden . 1975b. “Dispersal and Primary Establishment of Vegetation.” In Fennoscandian Tundra Ecosystems. Ecological Studies, edited by F. E. Wielgolaski , vol. 16, 82–85. Springer.

[ece371848-bib-0023] Erschbamer, B. , S. R. Niederfriniger , P. Carnicero , and R. Kaufmann . 2023. “Long‐Term Monitoring Confirms Limitations of Recruitment and Facilitation and Reveals Unexpected Changes of the Successional Pathways in a Glacier Foreland of the Central Austrian Alps.” Plant Ecology 224: 373–386. 10.1007/s11258-023-01308-2.

[ece371848-bib-0024] Faegri, K. 1967. The Plant World at Finse, Norway. University Botanical Museum.

[ece371848-bib-0025] Fernández‐Martínez, M. A. , S. Pérez‐Ortega , S. B. Pointing , et al. 2017. “Microbial Succession Dynamics Along Glacier Forefield Chronosequences in Tierra del Fuego (Chile).” Polar Biology 40, no. 10: 1939–1957. 10.1007/s00300-017-2110-7.

[ece371848-bib-0026] Fickert, T. 2020. “Common Patterns and Diverging Trajectories in Primary Succession of Plants in Eastern Alpine Glacier Forelands.” Diversity 12, no. 5: 191.

[ece371848-bib-0027] Fischer, A. , T. Fickert , G. Schwaizer , G. Patzelt , and G. Groß . 2019. “Vegetation Dynamics in Alpine Glacier Forelands Tackled From Space.” Scientific Reports 9, no. 1: 13918. 10.1038/s41598-019-50273-2. 31558792 PMC6763459

[ece371848-bib-0028] Flø, D. , and S. Hågvar . 2013. “Aerial Dispersal of Invertebrates and Mosses Close to a Receding Alpine Glacier in Southern Norway.” Arctic, Antarctic, and Alpine Research 45, no. 4: 481–490. 10.1657/1938-4246-45.4.481.

[ece371848-bib-0029] Frenot, Y. , B. Van Vliet‐Lanoë , and J.‐C. Gloaguen . 1995. “Particle Translocation and Initial Soil Development on a Glacier Foreland, Kerguelen Islands, Subantarctic.” Arctic and Alpine Research 27, no. 2: 107–115. 10.1080/00040851.1995.12003104.

[ece371848-bib-0030] Garrido‐Benavent, I. , S. Pérez‐Ortega , J. Durán , et al. 2020. “Differential Colonization and Succession of Microbial Communities in Rock and Soil Substrates on a Maritime Antarctic Glacier Forefield.” Frontiers in Microbiology 11: 126. 10.3389/fmicb.2020.00126. 32117148 PMC7018881

[ece371848-bib-0031] Giesen, R. , L. M. Andreassen , M. Van den Broeke , and J. Oerlemans . 2009. “Comparison of the Meteorology and Surface Energy Balance at Storbreen and Midtdalsbreen, Two Glaciers in Southern Norway.” Cryosphere 3: 57–74. 10.5194/tc-3-57-2009.

[ece371848-bib-0032] Giesen, R. , M. Van den Broeke , J. Oerlemans , and L. M. Andreassen . 2008. “Surface Energy Balance in the Ablation Zone of Midtdalsbreen, a Glacier in Southern Norway: Interannual Variability and the Effect of Clouds.” Journal of Geophysical Research 113: D21111. 10.1029/2008JD010390.

[ece371848-bib-0033] Girard, F. , S. Payette , and A. Delwaide . 2017. “Patterns of Early Postfire Succession of Alpine, Subalpine and Lichen‐Woodland Vegetation: 21 Years of Monitoring From Permanent Plots.” Forests 8: 346. 10.3390/f8090346.

[ece371848-bib-0034] Hågvar, S. 2012. “Primary Succession in Glacier Forelands: How Small Animals Conquer New Land Around Melting Glaciers.” In International Perspectives on Global Environmental Change, edited by S. Young and S. Silvern . IntechOpen. 10.5772/26536.

[ece371848-bib-0035] Hågvar, S. , and M. Gobbi . 2022. “The Role of Arthropods in Early Colonization Near Melting Glaciers: Contradictions Between Ecological Assumptions and Recent Study Results.” Acta Oecologica 114: 103820. 10.1016/j.actao.2022.103820.

[ece371848-bib-0036] Hågvar, S. , and M. Ohlson . 2013. “Ancient Carbon From a Melting Glacier Gives High 14C Age in Living Pioneer Invertebrates.” Scientific Reports 3, no. 1: 2820. 10.1038/srep02820. 24084623 PMC3788365

[ece371848-bib-0037] Hågvar, S. , M. Ohlson , and J. E. Brittain . 2016. “A Melting Glacier Feeds Aquatic and Terrestrial Invertebrates With Ancient Carbon and Supports Early Succession.” Arctic, Antarctic, and Alpine Research 48, no. 3: 551–562. 10.1657/AAAR0016-027.

[ece371848-bib-0038] Hauck, M. 2009. “Global Warming and Alternative Causes of Decline in Arctic‐Alpine and Boreal‐Montane Lichens in North‐Western Central Europe.” Global Change Biology 15: 2653–2661.

[ece371848-bib-0039] Haugland, J. E. , and S. W. Beatty . 2005. “Vegetation Establishment, Succession and Microsite Frost Disturbance on Glacier Forelands Within Patterned Ground Chronosequences.” Journal of Biogeography 32, no. 1: 145–153.

[ece371848-bib-0040] Heim, R. J. , A. Bucharova , L. Brodt , et al. 2021. “Post‐Fire Vegetation Succession in the Siberian Subarctic Tundra Over 45 Years.” Science of the Total Environment 760: 143425. 10.1016/j.scitotenv.2020.143425. 33172629

[ece371848-bib-0041] Herk, v. C. M. , A. Aptroot , and D. H. F. van Dobben . 2002. “Long‐Term Monitoring in The Netherlands Suggests That Lichens Respond to Global Warming.” Lichenologist 34, no. 2: 141–154.

[ece371848-bib-0042] Holt, E. A. , B. McCune , and P. Neitlich . 2006. “Defining a Successional Metric for Lichen Communities in the Arctic Tundra.” Arctic, Antarctic, and Alpine Research 38, no. 3: 373–377.

[ece371848-bib-0043] Inoue, T. , M. Uchida , M. Inoue , et al. 2019. “Vegetation Data of High Arctic Lichens on Austre Brøggerbreen Glacier Foreland, Ny‐Ålesund, Svalbard, in 1994.” Polar Data Journal 3: 1–11. 10.20575/00000005.

[ece371848-bib-0044] Jansson, P. 2008. “Literature Concerning the Tarfala Valley and its Close Surroundings (Tarfala Research Station Annual Report 2007/2008, Issue).”

[ece371848-bib-0045] Jansson, P. , G. Rosqvist , and P. Holmlund . 2008. “Monitoring, a Scientific Background. Monitoring at Tarfala Research Station.” *Tarfala Research Station*, Annual Report, 2007/2008, 1–14. https://www.su.se/natgeo/english‐old/tarfala‐research‐station/monitoring/monitoring‐a‐scientific‐background‐1.89884?cache=82%2Fpersonal.

[ece371848-bib-0046] Johansson, H. F. 1951. “Scientific Investigations in the Kebnekajse Massif, Swedish Lappland. II. The Petrology and Tectonics of the Kebnekajse Region and Their Morphological Importance.” Geografiska Annaler. Series A, Physical Geography 33, no. 1–2: 95–120.

[ece371848-bib-0047] Karlén, W. 1973. “Holocene Glacier and Climatic Variations, Kebnekaise Mountains, Swedish Lapland.” Geografiska Annaler: Series A, Physical Geography 55, no. 1: 29–63. 10.1080/04353676.1973.11879879.

[ece371848-bib-0048] Karlén, W. , and J. L. Black . 2002. “Estimates of Lichen Growth‐Rate in Northern Sweden.” Geografiska Annaler 84A, no. 3–4: 225–232.

[ece371848-bib-0049] Klopsch, C. , J. C. Yde , J. A. Matthews , A. E. Vater , and M. A. Gillespie . 2023. “Repeated Survey Along the Foreland of a Receding Norwegian Glacier Reveals Shifts in Succession of Beetles and Spiders.” Holocene 33, no. 1: 14–26. 10.1177/09596836221126032.

[ece371848-bib-0050] Koblet, T. , I. Gärtner‐Roer , M. Zemp , et al. 2010. “Reanalysis of Multi‐Temporal Aerial Images of Storglaciären, Sweden (1959–99)—Part 1: Determination of Length, Area, and Volume Changes.” Cryosphere 4, no. 3: 333–343. 10.5194/tc-4-333-2010.

[ece371848-bib-0051] Llambí, L. D. , A. Melfo , L. E. Gámez , et al. 2021. “Vegetation Assembly, Adaptive Strategies and Positive Interactions During Primary Succession in the Forefield of the Last Venezuelan Glacier.” Frontiers in Ecology and Evolution 9: 657755. 10.3389/fevo.2021.657755.

[ece371848-bib-0052] Losapio, G. , M. de la Cruz , A. Escudero , B. Schmid , and C. Schöb . 2018. “The Assembly of a Plant Network in Alpine Vegetation.” Journal of Vegetation Science 29, no. 6: 999–1006. 10.1111/jvs.12681.

[ece371848-bib-0053] Malanson, G. P. , D. B. Fagre , D. R. Butler , and Z. Shen . 2024. “Alpine Plant Communities and Current Topographic Microrefugia Vary With Regional Climates.” Geomorphology 458: 109241. 10.1016/j.geomorph.2024.109241.

[ece371848-bib-0054] Mallen‐Cooper, M. , E. Rodríguez‐Caballero , D. J. Eldridge , et al. 2023. “Towards an Understanding of Future Range Shifts in Lichens and Mosses Under Climate Change.” Journal of Biogeography 50: 406–417. 10.1111/jbi.14542.

[ece371848-bib-0055] Matthews, J. A. , and A. E. Vater . 2015. “Pioneer Zone Geo‐Ecological Change: Observations From a Chronosequence on the Storbreen Glacier Foreland, Jotunheimen, Southern Norway.” Catena 135: 219–230. 10.1016/j.catena.2015.07.016.

[ece371848-bib-0056] Matthews, J. A. , and R. J. Whittaker . 1987. “Vegetation Succession on the Storbreen Glacier Foreland, Jotunheimen, Norway: A Review.” Arctic and Alpine Research 19, no. 4: 385–395. 10.1080/00040851.1987.12002619.

[ece371848-bib-0057] Moreau, M. , D. Mercier , D. Laffly , and E. Roussel . 2008. “Impacts of Recent Paraglacial Dynamics on Plant Colonization: A Case Study on Midtre Lovenbreen Foreland, Spitsbergen (79 Degrees N).” Geomorphology 95, no. 1–2: 48–60. 10.1016/j.geomorph.2006.07.031.

[ece371848-bib-0058] Nascimbene, J. , H. Mayrhofer , M. Dainese , and P. O. Bilovitz . 2017. “Assembly Patterns of Soil‐Dwelling Lichens After Glacier Retreat in the European Alps.” Journal of Biogeography 44: 1393–1404. 10.1111/jbi.12970. 28701808 PMC5484317

[ece371848-bib-0059] Ndah, F. A. , A. Michelsen , R. Rinnan , M. Maljanen , S. Mikkonen , and M. Kivimäenpää . 2024. “Impact of Three Decades of Warming, Increased Nutrient Availability, and Increased Cloudiness on the Fluxes of Greenhouse Gases and Biogenic Volatile Organic Compounds in a Subarctic Tundra Heath.” Global Change Biology 30: e17416. 38994730 10.1111/gcb.17416

[ece371848-bib-0060] Østbye, E. 1997. Finseområdets Bibliografi 1781–1996 (The Bibliography of the Finse Area 1781–1996). Reports from the High Mountain Ecology Research Station, Finse.

[ece371848-bib-0061] Pastore, M. A. , A. T. Classen , A. W. D'Amato , J. R. Foster , and E. C. Adair . 2022. “Cold‐Air Pools as Microrefugia for Ecosystem Functions in the Face of Climate Change.” Ecology 103, no. 8: e3717. 10.1002/ecy.3717. 35388477

[ece371848-bib-0062] Phinney, N. H. , J. Asplund , and Y. Gauslaa . 2022. “The Lichen Cushion: A Functional Perspective of Color and Size of a Dominant Growth Form on Glacier Forelands.” Fungal Biology 126: 375–384. 10.1016/j.funbio.2022.03.001. 35501033

[ece371848-bib-0063] Prach, K. , J. Klimešová , J. Košnar , O. R. O. Redčenko , and M. Hais . 2012. “Variability of Contemporary Vegetation Around Petuniabukta, Central Spitsbergen.” Polish Polar Research 33, no. 4: 383–394. 10.2478/v10183-012-0026-z.

[ece371848-bib-0064] Rachlewicz, G. , W. Szczuciński , and M. Ewertowski . 2007. “Post‐‘Little Ice Age’ Retreat Rates of Glaciers Around Billefjorden in Central Spitsbergen, Svalbard.” Polish Polar Research 28: 159–186.

[ece371848-bib-0065] Reinardy, B. , A. Booth , A. Hughes , et al. 2019. “Pervasive Cold Ice Within a Temperate Glacier—Implications for Glacier Thermal Regimes, Sediment Transport and Foreland Geomorphology.” Cryosphere 13: 827–843. 10.5194/tc-13-827-2019.

[ece371848-bib-0066] Reinardy, B. T. I. , I. Leighton , and P. J. Marx . 2013. “Glacier Thermal Regime Linked to Processes of Annual Moraine Formation at Midtdalsbreen, Southern Norway.” Boreas 42, no. 4: 896–911. 10.1111/bor.12008.

[ece371848-bib-0067] Robbins, J. , and J. Matthews . 2009. “Pioneer Vegetation on Glacier Forelands in Southern Norway.” Journal of Vegetation Science 20: 889–902. 10.1111/j.1654-1103.2009.01090.x.

[ece371848-bib-0068] Rydgren, K. , R. Halvorsen , J. P. Töpper , and J. M. Njøs . 2014. “Glacier Foreland Succession and the Fading Effect of Terrain Age.” Journal of Vegetation Science 25: 1367–1380. 10.1111/jvs.12184.

[ece371848-bib-0069] Sancho, L. G. , D. Palacios , T. G. A. Green , M. Vivas , and A. Pintado . 2011. “Extreme High Lichen Growth Rates Detected in Recently Deglaciated Areas in Tierra del Fuego.” Polar Biology 34: 813–822. 10.1007/s00300-010-0935-4.

[ece371848-bib-0070] Šmilauer, P. , and J. Lepš . 2014. Multivariate Analysis of Ecological Data Using CANOCO 5. 2nd ed. Cambridge University Press. 10.1017/CBO9781139627061.

[ece371848-bib-0071] Sørlie, R. 2001. “Ectomycorrhiza on *Salix herbacea* L. in the Glacier Foreland of Midtdalsbreen, Finse, Norway. 1.”

[ece371848-bib-0072] Spribille, T. , A. M. Fryday , S. Pérez‐Ortega , et al. 2020. “Lichens and Associated Fungi From Glacier Bay National Park, Alaska.” Lichenologist 52, no. 1: 61–181. 10.1017/S0024282920000079. 32788812 PMC7398404

[ece371848-bib-0073] Stavrova, N. I. , V. V. Gorshkov , P. N. Katjutin , and I. J. Bakkal . 2020. “The Structure of Northern Siberian Spruce–Scots Pine Forests at Different Stages of Post‐Fire Succession.” Forests 11: 588. 10.3390/f11050558.

[ece371848-bib-0074] Stork, A. 1963. “Plant Immigration in Front of Retreating Glaciers, With Examples From the Kebnekajse Area, Northern Sweden.” Geografiska Annaler 45, no. 1: 1–22.

[ece371848-bib-0075] Timoshok, E. E. , E. N. Timoshok , I. I. Gureyeva , and S. N. Skorokhodov . 2020. “Primary Successions of Vegetation on the Young Moraines in the Severo‐Chuiskiy Center of Glaciation (Central Altai).” Contemporary Problems of Ecology 13, no. 1: 36–47. 10.1134/S1995425520010114.

[ece371848-bib-0076] Vanneste, T. , O. Michelsen , B. J. Graae , et al. 2017. “Impact of Climate Change on Alpine Vegetation of Mountain Summits in Norway.” Ecological Research 32, no. 4: 579–593. 10.1007/s11284-017-1472-1.

[ece371848-bib-0077] Vargas, C. R. 2018. “Lichens on the Edge: Studying the Lichens at the Union Glacier.” Advances in Chilean Antarctic Science 4: 11–12.

[ece371848-bib-0078] Vetaas, O. R. 1994. “Primary Succession of Plant Assemblages on a Glacier Foreland‐Bødalsbreen, Southern Norway.” Journal of Biogeography 21: 297–308. 10.2307/2845531.

[ece371848-bib-0079] Wietrzyk, P. , K. Rola , P. Osyczka , P. Nicia , W. Szymański , and M. Węgrzyn . 2018. “The Relationships Between Soil Chemical Properties and Vegetation Succession in the Aspect of Changes of Distance From the Glacier Forehead and Time Elapsed After Glacier Retreat in the Irenebreen Foreland (NW Svalbard).” Plant and Soil 428: 195–211. 10.1007/s11104-018-3660-3.

[ece371848-bib-0080] Wietrzyk‐Pełka, P. , V. Otte , M. H. Węgrzyn , and M. Olech . 2018. “From Barren Substrate to Mature Tundra—Lichen Colonization in the Forelands of Svalbard Glaciers.” Acta Societatis Botanicorum Poloniae 87, no. 4: 3599.

[ece371848-bib-0081] Wietrzyk‐Pełka, P. , K. Rola , A. Patchett , W. Szymański , M. H. Węgrzyn , and R. G. Björk . 2021. “Patterns and Drivers of Cryptogam and Vascular Plant Diversity in Glacier Forelands.” Science of the Total Environment 770: 144793. 10.1016/j.scitotenv.2020.144793. 33497901

